# Fault Detection and Isolation Based on Structural Analysis: Application to a Multi-Engine Propulsion Cluster

**DOI:** 10.3390/s25041054

**Published:** 2025-02-10

**Authors:** Renato Murata, Julien Marzat, Hélène Piet-Lahanier, Sandra Boujnah, Pierre Belleoud

**Affiliations:** 1 Département Traitement de l’Information et des Systèmes (DTIS), Office National d’Études et de Recherches Aérospatiales (ONERA), Université Paris-Saclay, 6 Chem. de la Vauve aux Granges, 91120 Palaiseau, France; renato.murata@onera.fr (R.M.); helene.piet-lahanier@onera.fr (H.P.-L.); 2 Centre National d’Études Spatiales (CNES), Sous-Direction Techniques Systèmes de Transport Spatial, 52 Rue Jacques Hillairet, 75012 Paris, France; sandra.boujnah@cnes.fr (S.B.); pierre.belleoud@cnes.fr (P.B.)

**Keywords:** multi-engine cluster, residual selection, residual sensitivity, fault signature

## Abstract

A multi-engine cluster of a launcher operates in extreme conditions, making the tasks of detection and isolation complex, with only a precise group of faults with small magnitudes worth being detected and isolated. To address this, a model-based Fault Detection and Isolation (FDI) system is developed for a multi-engine cluster using Structural Analysis (SA). A model of a multi-engine cluster composed of three engines is established, where different sensor and actuator faults are considered. After analyzing the model using SA, more than 16,000 residual generator candidates are obtained. An algorithm is then proposed to find subsets of residuals with minimal cardinality capable of isolating all faults. Each subset generates a unique fault signature for each fault, enabling fault isolation. The selection of the best subsets is based on the new definition of the Subset Sensitivity Index (SSI), according to which the subsets composed of the most sensitive residuals have the highest SSI values. The residual sensitivity is calculated using a new method based on the signal-to-noise ratio (SNR), where a new definition of the Residual Sensitivity Index (RSI) is given. Furthermore, a new algorithm is presented for fault isolation. Suppose a theoretical fault signature is not observed. In that case, the algorithm uses the knowledge of the residual sensitivity (RSI) to define which residual did not behave as expected and predicts which fault is active in the system. Monte Carlo simulations are conducted under ten different faults to analyze the performance of the proposed FDI system, including actuators and sensor faults.

## 1. Introduction

Efficient fault detection and isolation systems for launchers are a major requirement. However, the development of such systems is an inherently complex task due to the unique conditions under which these vehicles operate. The environment includes extremely high and low temperatures, intense vibrations, and rapid changes in operating conditions, which makes the accurate measurement of those variables difficult. Unlike other applications, weight and space constraints limit the instrumentation used to determine the health state of the system. The development of Health Monitoring Systems (HMSs), which includes the Fault Detection and Isolation (FDI) task, has been a subject of research since the early 1970s [1]. The first strategy consisted of examining if the measurement-critical parameters were within a safe limit. If the parameters were outside safe limits, the HMS would trigger alerts or automatic shutdown.

The importance of an FDI system was brought to another level with the development of new Reusable Launch Vehicles (RLVs) in the mid-2010s. In contrast to expendable launch vehicles, RLVs are designed to be launched multiple times. In order to ensure the vehicle’s reusability, a high-performance FDI system is necessary. Furthermore, sophisticated technologies are incorporated into RLVs to enable its recovery, which allows for the implementation of powerful FDI systems. RLVs have new requirements for thrust control compared to traditional vehicles, particularly during vertical landings, when only a portion of the thrust is needed [2]. One possible solution is the use of a multi-engine cluster [3]. In addition to the large range of thrust modulation, a multi-engine cluster also offers higher mission reliability when compared with a single engine [3]. If a fault occurs, e.g., loss of thrust or extinction, in one engine, the remaining healthy engines can be used to attenuate the effect of this fault [4]. In order to attenuate the effect of a fault, an FDI system is necessary.

There are many methods for developing an FDI system. In [5], the methods were classified into three main categories: quantitative model-based, qualitative model-based, and data-based methods. Recent studies addressing the FDI problem in launchers have mostly relied on quantitative model-based and data-based methods. The quantitative methods are mainly linked to state observers [6,7,8,9], especially variations of Kalman filters [10,11,12,13,14]. The main drawback of these methods is that the launcher’s model is very complex, with numerous nonlinear equations. Computing a state observer for the entire launcher ca,n thus, be very challenging. Concerning data-based methods, machine learning techniques [15,16,17], particularly neural networks [18,19,20,21,22,23,24,25], have dominated recent contributions to FDI systems in the context of launchers. However, implementing these machine learning techniques requires a significant amount of training data, which are extremely difficult to obtain because launch vehicles are strategic assets for industries and nations and are not available during the early development phase. A significant gap is observed in the open literature: the qualitative model-based approach has rarely been used in the development of FDI systems for launch vehicles.

This paper addresses the development of a model-based FDI system for a reusable launcher using a qualitative model-based approach based on Structural Analysis (SA). Even with the recent development of SA for FDI systems [26,27,28,29], to the best of our knowledge, only one paper [30] has employed SA for fault diagnosis in rocket engines. In [30], a specific subsystem of the rocket engine was considered: the LOX turbopump. The paper addressed the problem of estimating the fault magnitude. The considered faults were parametric deviations in the efficiency of the turbopump. They affected the dynamic equation that describes the evolution of the pump shaft speed. The model has six equations, and SA was used to find independent subsystems. Fault diagnosis filters were calculated for each subsystem and provided an accurate estimation of the faults. However, the study considered a rocket’s engine subsystem composed of six equations. In contrast, a multi-engine cluster model can contain over 100 equations. The number of equations directly impacts the complexity of an FDI system when using structural analysis because, in large-scale systems, the number of subsystems derived from SA increases exponentially. Thus, it becomes necessary to establish selection criteria to determine which subsystems will be utilized.

**Figure 1:**
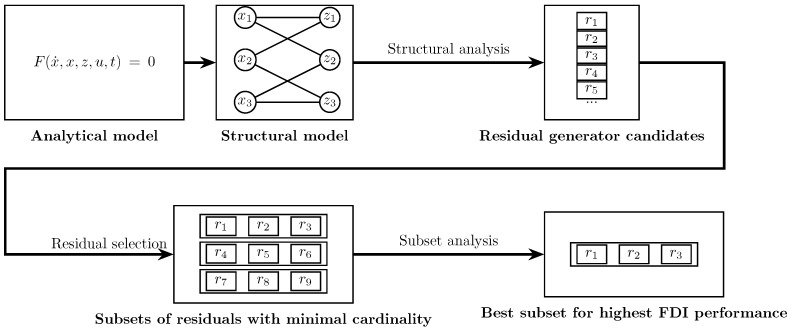
Proposed fault detection and isolation scheme.

A scheme summarizing the FDI system proposed here is illustrated in Figure 1. It can be divided into five main steps:

1. Analytical model development: The analytical model of the system is derived from differential and algebraic equations.
2. Structural model acquisition: The structural model is obtained from the analytical model.
3. Structural analysis: The structural model is used to compute residual generator candidates through SA.
4. Residual selection: Subsets of residuals with minimal cardinality, capable of detecting and isolating all considered faults, are formed using a residual selection algorithm.
5. Subset analysis: The subset that offers the best FDI performance is selected

Contributions concerning Steps 1, 2, and 3 were addressed in our previous works in a less complex scenario, where only a small range of faults was considered. In [31], SA was applied in a multi-engine cluster to investigate the possibility of fault detection and isolation, considering different measurement scenarios.

The residual selection algorithm was first introduced in [32]. The residual selection problem has been addressed by other authors [33,34]. However, in both cases, the isolation constraint was too restrictive, and the cardinality of the subsets was not minimal. The selection algorithm we propose relies on a weaker isolation constraint based on the fault signature. It returns subsets of residual generators with minimal cardinality capable of producing a unique fault signature.

To achieve efficient fault detection and isolation of faults, a possible approach is to search for the subset of residuals with the highest fault sensitivity. To quantify the sensitivity of a subset to faults, the Subset Sensitivity Index (SSI) is introduced. The subsets with the highest SSI values are considered the most effective for fault detection and isolation. The SSI is calculated based on the Residual Sensitivity Index (RSI), which measures the sensitivity of a residual to a specific fault. The RSI is calculated using the signal-to-noise ratio (SNR), and the residual signal in the absence of faults is used as the baseline for noise.

Fault isolation is performed by analyzing the fault signature. If a perfect match between the theoretical fault signature and the observed fault signature is not observed, a new method is proposed to isolate the most probable fault present in the system. One common method is to use the closest fault signature, as described in [35] and applied in [36] for fault isolation in wind turbines. The main drawback is that, depending on the observed fault signature, it may return a list of probable faults. The problem of finding the most probable fault was also addressed in [37], where a ‘consistency index’ was used. The index is calculated using the probability distribution of the residual signal by quantifying how confident the signal is in its nominal (or fault-free) condition. However, it does not account for the different fault sensitivities of the residuals. A method that considers the numerical fault sensitivity was proposed in [38], where the sensitivity of the residuals was calculated considering the partial derivative of the residual with respect to the fault. While powerful, this solution requires the analytical expression of the residuals. Inspired by the solution proposed in [38], we also propose the use of the RSI, which was first calculated to select the most sensitive subsets, for fault isolation to identify the most probable fault present in the system.

The main contributions of this paper include a comprehensive FDI technique based on structural analysis, which spans from the qualitative analysis of the analytical model to fault isolation. The technique includes a new method to evaluate subsets of residuals and determines which subsets offer the highest FDI performance. In addition, a new fault isolation technique is proposed, based on the predefined sensitivity of the residuals to faults. Finally, these methods are applied to a complete model of a multi-engine cluster for the first time. The findings discussed here were originally developed as part of the author’s thesis manuscript [39].

The remainder of this paper is organized as follows. In Section 2, the multi-engine cluster model under study is presented. Structural analysis of the model is performed in Section 3. The residual selection algorithm is recalled in Section 4. The numerical residual sensitivity is calculated in Section 5. Section 6 presents the fault isolation methods. Finally, the proposed methods are applied to the multi-engine cluster model in Section 7. Conclusions and perspectives are presented in Section 8 and Section 9, respectively.

## 2. Multi-Engine Cluster Model

**Figure 2:**
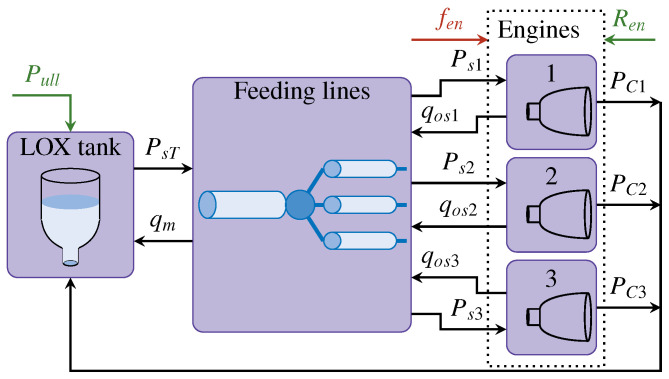
Multi-engine cluster scheme.

The multi-engine cluster model considered here was first presented in [31] with a smaller range of possible faults. It considers the LOX side of the cluster and comprises three subsystems: a LOX tank, feeding lines, and rocket engines. The cluster contains three identical Liquid-Propellant Rocket Engines (LPREs). A scheme of the simulation model of the cluster, containing all subsystems and the interactions between them, is illustrated in Figure 2.

Each rocket engine (i∈[1,3]) has its own control system. The control system receives a thrust reference ($R_{en}$) and uses the engine’s control valves to manage the pressure in the combustion chamber $\left(P_{Ci}\right)$. The pressure in the combustion chamber has a linear relation with the generated thrust. According to the control-valve opening surface, a LOX mass flow is used by the engines ($q_{osi}$); this mass flow is imposed as the outlet mass flow of the feeding lines. Knowing the mass flow of each line, the total mass flow that leaves the LOX tank ($q_{m}$) can be defined. The LOX pressure at the output of the tank ($P_{sT}$) depends on the ullage pressure ($P_{ull}$) and the hydrostatic pressure. The hydrostatic pressure depends on the rocket’s acceleration, which is directly related to the thrust generated by the engines and, therefore, the pressure in the combustion chamber ($P_{Ci}$). The outlet LOX tank pressure ($P_{sT}$) is imposed as the input pressure of the feeding lines. The outlet pressure of the feeding lines ($P_{si}$) is imposed as the input pressure of the engine’s turbopumps. All considered faults ($f_{en}$) affect the rocket engines.

### 2.1. Liquid-Propellant Rocket Engine

The equations of the LPRE model were obtained from [40] (Chapter 4). In the model, the LPRE system generates thrust by expelling hot gases from the combustion chamber, as represented by the chamber pressure ($P_{Ci}$). The combustion chamber receives propellants through two lines for oxidizer and fuel. The propellants are provided by two turbopumps—one for the oxidizer and another for the fuel. The turbopumps are powered using the exhaust gas from a small combustion chamber called the gas generator. Four electro-valves control the oxygen and fuel flow that enters both combustion chambers. A fifth valve is used to direct the distribution of the exhaust gas from the gas generator between oxygen and fuel turbines.

The equations of the LOX side of the cluster are presented here. The equations on the ${LH}_{2}$ side have the same structure, with the $._{O}$ index replaced by $._{H}$. The outlet pressure of the LOX turbopump ($P_{pOi}$) is express using manufacturer data as follows:

(1) $$\begin{array}{cc} P_{pOi}=\left(\frac{ap_{O}}{\rho_{O}}+R_{OGC}\right){\left(q_{COi}+q_{GOi}\right)}^{2}\\ & +\;bp_{O}\left(q_{COi}+q_{GOi}\right)\omega_{Oi}+cp_{O}\rho_{O}\omega_{Oi}^{2}. \end{array}$$

The evolution of the oxygen mass flow that enters the combustion chamber ($q_{COi}$) is derived from the momentum balance equation. The effect of the fault is highlighted in red:

(2) $$\begin{array}{cc} {\dot{q}}_{COi}=\frac{1}{I_{CO}}\left[P_{si}\right.+P_{pOi}-P_{Ci}\\ & -\left(\frac{1}{2\rho_{O}{\left(V_{COi}+\begin{array}{c} f_{VCOi} \end{array}\right)}^{2}}+R_{OC}+R_{OGC}\right)q_{COi}^{2}]. \end{array}$$

The LOX mass flow that enters the gas generator ($q_{GOi}$) is also derived from the conservation of momentum equation:

(3) $$\begin{array}{cc} {\dot{q}}_{GOi}=\frac{1}{I_{GO}}[P_{si}+P_{pOi}-P_{Gi}\\ & -\left(\frac{1}{2\rho_{O}{\left(V_{GOi}+\begin{array}{c} f_{VGOi} \end{array}\right)}^{2}}+R_{OG}+R_{OGG}\right)q_{GOi}^{2}] \end{array}$$

The hot gas mass flows that provide energy to the turbines are given by

(4) $$\begin{array}{c} {\dot{q}}_{THi}=\frac{1}{I_{TH}}\left(P_{Gi}-P_{THi}\right)-ky_{TH}R_{outG}\frac{T_{G}}{P_{Gi}}q_{THi}^{2} \end{array}$$

(5) $$\begin{array}{c} {\dot{q}}_{TOi}=\frac{1}{I_{TO}}\left(P_{Gi}-P_{TOi}\right)-Zr_{i}R_{outGG}\frac{T_{G}}{P_{Gi}}q_{TOi}^{2} \end{array}$$

where $Zr_{i}$ is the equivalent resistive coefficient of the valve. According to (5), valve $Zr_{i}$ is used to control the hot mass flow of the LOX turbine, while the resistive coefficient of the mass flow of the ${LH}_{2}$ turbine is fixed at a constant value of $ky_{TH}$ (4).

The evolution of the combustion chamber pressure ($P_{Ci}$) can be approximated by first-order Taylor expansion.

(6) $$\begin{array}{c} {\dot{P}}_{Ci}=k1_{C}\left(q_{CHi}+q_{fCOi}\right)-k2_{C}\sqrt{T_{C}}P_{Ci} \end{array}$$

(7) $$\begin{array}{c} q_{fCOi}=q_{COi}-\begin{array}{c} f_{qCOi} \end{array}. \end{array}$$

The gas generator pressure ($P_{Gi}$) has the same structure; the main difference is that no leakage is considered:

(8) $$\begin{array}{cc} \; & {\dot{P}}_{Gi}=k1_{G}\left(q_{GHi}+q_{GOi}\right)-k2_{G}\sqrt{T_{G}}P_{Gi}-k3_{G}\left(q_{TOi}+q_{THi}\right) \end{array}$$

The oxygen turbine pressure ($P_{TOi}$) is defined as

(9) $${\dot{P}}_{TOi}=k1_{TO}T_{G}q_{TOi}-k2_{TO}\sqrt{T_{G}}P_{TOi}.$$

The rotational speed’s evolution is expressed using manufacturer data as follows:

(10) $$\begin{array}{cc} {\dot{\omega }}_{Oi}=\frac{1}{J_{O}}[T_{TOi}-\frac{ac_{O}}{\rho_{O}}{\left(q_{COi}+q_{GOi}\right)}^{2}\\ & -bc_{O}\left(q_{COi}+q_{GOi}\right)\omega_{Oi}-cc_{O}\rho_{O}\omega_{Oi}^{2}] \end{array}$$

where the motor torque ($T_{TOi}$) is given by $T_{TOi}=ST.W_{i}$, with ST representing the specific torque and $W_{i}$ representing the work provided by the turbine pressure ($P_{TOi}$).

### 2.2. Feeding Lines

The feeding line model of the LOX side of a rocket with three engines was introduced in [41]. The optimal configuration in terms of minimal mass and pressure-drop values is a main line splitting into three secondary lines [42], as illustrated in Figure 2. This configuration consists of a total of four pipes. The model of one generic rigid pipe, considering dynamic compressibility and perfect thermal isolation and neglecting fluid thermal expansion, is defined by a set of two equations [43]:

(11) $$\begin{array}{ccc} \; & \; & \begin{array}{cc} \; & \mathrm{Momentum}\;\mathrm{balance}:\\ \; & \dot{q}=\frac{S}{L}\left(P_{in}-P-\frac{f_{r}L}{2\rho S^{2}D}q^{2}\right) \end{array} \end{array}$$

(12) $$\begin{array}{ccc} \; & \; & \begin{array}{cc} \; & \mathrm{Mass}\;\mathrm{balance}:\\ \; & \dot{P}=\frac{\alpha^{2}}{V}\left(q-q_{o}\right) \end{array} \end{array}$$

where $P_{in}$ is the inlet pressure of the pipe, *P* is the outlet pressure, *q* is the inlet mass flow, and $q_{o}$ is the outlet mass flow. Equations (11) and (12) are repeated for each of the four pipes, and the $._{m}$ and $._{si},i\in \left[1,3\right]$ indices are used to denote the main and secondary lines, respectively. The inlet pressure of the main line is equal to the output pressure of the tank ($P_{in,m}=P_{sT}$), and the inlet pressure of the secondary lines is equal to the outlet pressure of the main line ($P_{si}=P_{m}$). The outlet mass flow of the main line is the sum of mass flows of each secondary line ($q_{om}=q_{s1}+q_{s2}+q_{s3}$). Finally, the outlet mass flow of the secondary lines is the sum of the LOX mass flow used by the gas generator and the combustion chamber; for secondary line 1, for instance, $q_{os1}=q_{GO1}+q_{CO1}$.

### 2.3. LOX Tank

A simple equation is used to estimate the outlet pressure of the LOX tank ($P_{sT}$). The equation depends on the ullage pressure and the hydrostatic pressure [44]:

(13) $$P_{sT}=P_{ull}+\rho_{O}\left[a_{L}+g\cos\left(b\right)\right]H_{d}$$

where $a_{L}$ and *g* are the acceleration of the rocket and of gravity, respectively; $H_{d}$ is the gravitational head; and *b* is the inclination angle. The gravitational acceleration is considered to be constant. The gravitational head can be estimated by knowing the remaining volume of fluid ($V_{LOX}$) and the tank’s geometry. Considering that a cylinder can approximate the shape of the tank, the gravitational head ($H_{d}$) is defined as

(14) $$H_{d}=\frac{V_{LOX}}{\pi r^{2}},\;V_{LOX}=V_{LOX_{0}}-\frac{\int_{0}^{t}q_{m}\;dt}{\rho_{0}}$$

where $V_{LOX_{0}}$ is the initial volume of the LOX in the tank.

The rocket’s acceleration ($a_{L}$) can be approximated by a bivariate quadratic function of the total thrust (*T*) generated by the engines and the mass of the rocket ($m_{R}$):

(15) $$a_{L}=k_{1a}+k_{2a}T+k_{3a}m_{R}+k_{4a}Tm_{R}+k_{5a}m_{R}^{2}$$

where constants $k_{ja}$ and j∈[1,5] are related to the rocket’s aerodynamics. Considering a linear relation between the thrust and the pressure in the combustion chamber, the thrust provided by engine 1 can be expressed as $T_{i}=k_{T}P_{C1}$, where $k_{T}$ is a constant. The total thrust is the sum of the thrust generated by each engine $T=k_{T}\sum_{i=1}^{3}P_{Ci}$. Finally, the mass of the rocket ($m_{R}$) can be calculated as follows:

(16) $$m_{R}=m_{R_{0}}-\int_{0}^{t}q_{m}\;dt-\int_{0}^{t}q_{H}\;dt$$

where $q_{H}=q_{CHi}+q_{GHi}$ is the total hydrogen mass flow used by the three engines and $m_{R_{0}}$ is the initial mass of the rocket.

### 2.4. Fault Injection

A set of actuators and sensor faults is considered in this study. All considered faults are described in Table 1.

Control-valve faults affect the evolution of the mass flow in the combustion chamber and gas generator, as explained in Equations (2) and (3), respectively. LOX leakage affects the pressure in the combustion chamber (Equations (6) and (7)). The considered sensor faults are all additive faults that affect the measurement equation. For instance, the measurement of the rotating speed of the *i*-th LOX turbopump is defined as follows:

(17) $$y\omega_{Oi}=\omega_{Oi}+\begin{array}{c} f_{wOi} \end{array}+\delta_{Oi}$$

where $\delta_{Oi}$ is the measurement noise. Sensor faults $\begin{array}{c} f_{RMCi} \end{array}$ and $\begin{array}{c} f_{RMGi} \end{array}$ affect the measurement of the mixture ratio in the combustion chamber and gas generator, respectively. The mixture ratio in both chambers is defined as follows:

(18) $$\begin{array}{cc} yRMC_{i}=\frac{q_{fCOi}}{q_{CHi}}+\begin{array}{c} f_{RMCi} \end{array}+\delta_{RMCi} & yRMG_{i}=\frac{q_{GOi}}{q_{GHi}}+\begin{array}{c} f_{RMG1} \end{array}+\delta_{RMGi}. \end{array}$$

## 3. Structural Analysis

In this section structural analysis is performed for the multi-engine cluster model. The analysis returns a large number of residuals that would be infeasible to implement. An algorithm to select the best residuals is presented in Section 4; it performs a qualitative analysis of the residuals and returns subsets of residuals with minimal cardinality capable of isolating all faults. In order to select the subsets that use the most sensitive residuals, a method that performs a quantitative analysis of the residuals is presented in Section 5. Section 6 presents a fault isolation algorithm that considers residual sensitivity. Finally, the combination of these methods is applied in Section 7 to a multi-engine cluster case study, where simulation results are presented.

To perform the structural analysis of the model presented in Section 2, the structural model must be constructed. This model can be extracted from a system described by nonlinear differential–algebraic equations of the following form:

(19) $$V(\dot{X},X,Z,F,t)=0$$

where *X* is the vector of unknown variables in $R^{ns}$ and its derivatives ($\dot{X}$), *Z* is the vector of known variables in $R^{nz}$, *F* is the fault vector in $R^{nf}$, *t* is the time, and *V* is the vector of 2ns+nz algebraic equations. One requirement to build the structural model is that each fault (f∈F) must affect only one equation from *V*. This requirement is easily fulfilled with intermediate variables if one fault simultaneously impacts more than one equation.

A large range of systems can be written in the form described in (19). The multi-engine model can be written in this form by defining the known vector (*Z*) as the concatenation of the input vector (*u*) and output measurement vector (*y*), which yields Z={u,y}. Only variables related to rocket engine 1 are considered for further analysis, since all results obtained for engine 1 are valid for the other two engines due to the symmetry of the engine cluster. The vector of unknown variables (*X*), input variables (*u*), output measurements (*y*), and the fault vector (*F*) are defined as follows:

(20) $$\begin{array}{cc} \; & \left\{\begin{array}{cc} X= & [P_{m},P_{s1},q_{s1},q_{os1},P_{pO1},P_{pH1},q_{CO1},q_{fCO1},q_{GO1},q_{CH1},q_{GH1},q_{TH1},q_{TO1},\\ \; & P_{C1},P_{G1},P_{TO1},P_{TH1},\omega_{O1},\omega_{H1},RMC_{1},RMG_{1},P_{sT},a_{L},m_{R1}{]}^{T} \end{array}\right. \end{array}$$

(21) $$\begin{array}{cc} \; & \left\{\begin{array}{c} u={\left[V_{GO1},V_{GO1},V_{GH1},V_{CH1}\right]}^{T}\\ y={\left[yP_{m},yP_{s1},yP_{G1},yP_{C1},y\omega_{O1},y\omega_{H1},yRMC_{1},yRMG_{1}\right]}^{T}\\ Z={\left[u,y\right]}^{T} \end{array}\right. \end{array}$$

(22) $$\begin{array}{cc} \; & \left\{\begin{array}{c} F={\left[f_{VCH1},f_{VCO1},f_{VGH1},f_{VGO1},f_{qCO1},f_{RMC1},f_{RMG1},f_{PC1},f_{\omega_{O1}},f_{\omega_{H1}}\right]}^{T}. \end{array}\right. \end{array}$$

Using the vectors defined in Equations (20)–(22), the equations presented in Section 2 are used to build the structural model. The equations from the LOX tank presented in Section 2.3 were not used in the structural analysis because they are affected by the faults from engines 2 and 3, which are not addressed here.

The structural model is a bipartite graph that abstracts the form of the equations and retains only information for which variables are present in the equation. Let *W* represent all the variables from (19), i.e., $W=\{\dot{X},X,Z,F,t\}$, and the structural model of (19) is defined as follows [45]:

**Definition** **1**. *Structural model: The structural model of the system (V,W) is a bipartite graph (V,W,E) where E⊂V×W is the set of edges defined as follows:*

*$(V_{j},W_{k})\in E$ if the $W_{k}$ variable appears in equation $V_{j}$.*

### 3.1. Dulmage–Mendelsohn Decomposition

The structural model determines which faults can be detected and isolated using the measurement assumptions from Section 2. To do so, the Dulmage–Mendelsohn (DM) decomposition [46] of the incidence matrix is calculated using the Fault Diagnosis Toolbox [28]. Decomposition rearranges the system’s equations (*V*) into three parts: the underdetermined part ($V^{-}$), where the number of equations is smaller than the number of unknown variables; the exactly determined part ($V^{0}$), where the number of equations is equal to the number of unknown variables; and the overdetermined part ($V^{+}$), where the number of equations is larger than the number of unknown variables. Only the faults that affect the equations localized at the overdetermined part of the model can be detected, since it is the part of the system that can generate residuals.

**Figure 3:**
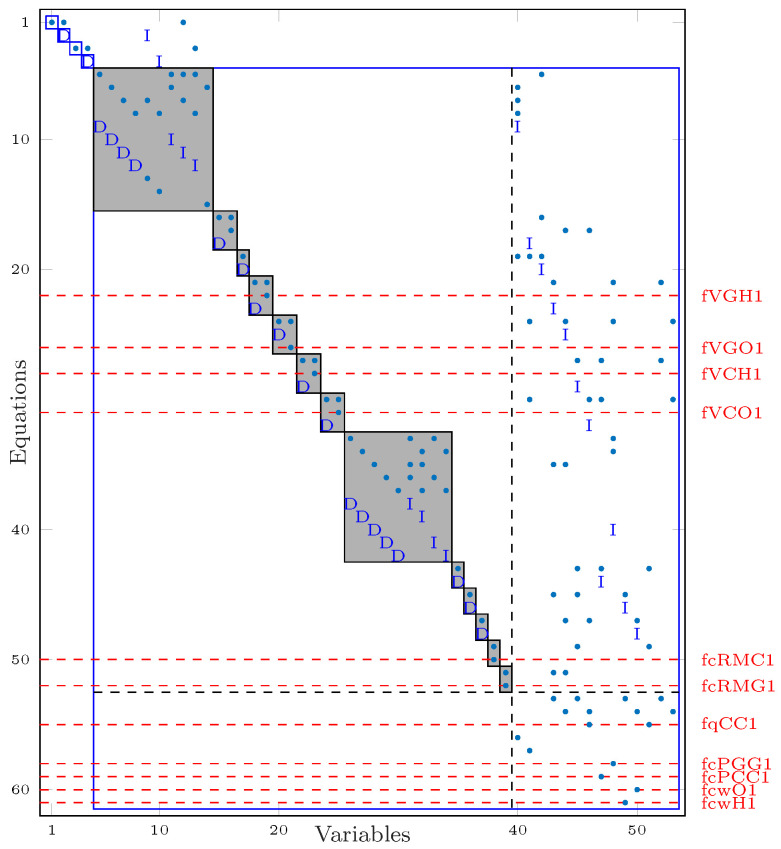
Dulmage–Mendelsohn decomposition.

DM decomposition is depicted in Figure 3. The equations in the overdetermined part are confined within the large blue rectangle. All considered faults impact equations localized in the overdetermined part and can, thus, be detected. The structural isolability of the faults is defined by the gray rectangles called equivalent classes. A group of equations in the same equivalent class is necessary to build an overdetermined set, i.e., two faults affecting the same equivalent class cannot be structurally isolatable. From Figure 3, the selected faults can be isolated, with each one affecting different equivalent classes.

### 3.2. Residual Generator Candidates

Structural analysis allows for the systematic computation of residual generator candidates. An ideal residual generator is defined as follows.

**Definition** **2** (Ideal residual generator)**.** *Consider a model (V) such as (19). A system (R) with input Z and output r is a residual generator for V and r is a residual if f=0 implies r=0 for all f∈F.*

In practice, residuals are not ideal due to measurement noise and model uncertainties, but they have a value close to zero. Each residual is affected by a predefined group of faults, which determines its sensitivity. The fault sensitivity of a residual generator ($R_{i}$) is defined below.

**Definition** **3** (Fault sensitivity)**.** *Let $R_{i}$ be a residual generator for the model (V) (19). Then, $R_{i}$ is sensitive to fault f∈F if f≠0 implies $r_{i}\neq 0$.*

From a structural analysis perspective, Minimally Structurally Overdetermined (MSO) setsare residual generators. The computation of MSO sets is possible if the degree of redundancy of a system is larger than one. The degree of redundancy of the system (19) is defined as follows:

(23) $$\phi \left(V\right)=|V^{+}\left|-\right|W_{x}^{+}|.$$

where $|V^{+}|$ and $|W_{x}^{+}|$ are the numbers of equations and unknown variables in the overdetermined part of the system, respectively. The degree of redundancy is directly related to the number of measurements. Each added measurement increases the number of equations while the number of unknown variables remains constant, raising the degree of redundancy.

An efficient method to find MSO sets [47] is implemented by the fault diagnosis toolbox. The formal definition of the MSO set obtained in [48] is recalled below.

**Definition** **4**. *A non-empty set of equations ($F_{b}\neq ⌀$) is a Proper Structurally Overdetermined (PSO) set if $F_{b}=F^{+}$, where $F^{+}$ is the set of equations of F in the overdetermined part.*

**Definition** **5**. *A PSO set is a minimally structurally overdetermined set if no proper subset is a PSO set.*

According to Definition 5, every MSO set has one more equation than unknown variables. An MSO set is considered a candidate for a residual generator because it is a testable submodel that can be solved independently. In addition, it has one extra equation that can be used to generate a residual signal.

The multi-engine cluster has a degree of redundancy of 8, with 53 unknown variables and 61 equations in the overdetermined part of the system. The number of MSO sets increases exponentially with the degree of redundancy of the system. In total, 16441 MSO sets are computed for the engine cluster. To illustrate the form of an MSO, all equations used by MSO 172 (denoted $R_{172}$) are listed below.

(24) $$\begin{array}{c} \begin{array}{cc} \; & R_{172}\;\mathrm{equations}:\;\\ \; & {\dot{q}}_{GO1}\left(P_{G1},P_{pO1},P_{s1},V_{GO1},f_{VGO1},q_{GO1}\right)\\ \; & {\dot{q}}_{CO1}\left(P_{C1},P_{pO1},P_{s1},V_{CO1},f_{VCO1},q_{CO1}\right)\\ \; & {\dot{P}}_{C1}\left(q_{CH1},q_{fCO1},P_{C1}\right)\\ \; & \dot{\omega_{O1}}\left(\omega_{O1},q_{CO1},q_{GO1}\right)\\ \; & P_{PO1}\left(q_{CO1},q_{GO1},\omega_{O1}\right) \end{array} \end{array}$$

(25) $$\begin{array}{c} \begin{array}{cc} \; & R_{172}\;\mathrm{known}\;\mathrm{variables}:\;\\ \; & Z_{172}=\left[yP_{s1},yP_{G1},yRMC_{1},y\omega_{O1},V_{CO1},V_{GO1}\right]. \end{array} \end{array}$$

(26) $$\begin{array}{c} \begin{array}{cc} \; & R_{172}\;\mathrm{sensitivity}:\;\\ \; & R_{172,f}=\left[f_{VGO1},f_{VCO1},f_{qCO1},f_{RMC1},f_{\omega_{O1}}\right]. \end{array} \end{array}$$

(27) $$\begin{array}{c} \begin{array}{cc} \; & R_{172}\;\mathrm{residual}\;\mathrm{signal}:\;\\ \; & r_{172}=y\omega_{O1}-\omega_{O1} \end{array} \end{array}$$

The analytical expressions of Equation (24) are given in Equations (1)–(3), (6) and (10). Analyzing the set of equations and known variables used by $R_{172}$, it is possible to notice how this group of equations can be solved independently. For instance, the LOX mass flow in the gas generator (${\dot{q}}_{GO1}$) depends on the pressure in the gas generator ($P_{G1}$). Instead of using the expression of $P_{G1}$ defined in Equation (8), the direct measurement of $P_{G1}$ ($yP_{G1}$) is employed. The same happens with the ${LH}_{2}$ combustion chamber mass flow ($q_{CH1}$), which is estimated directly from the measurement of $RMC_{1}$. In addition, using measurements to replace the analytical expression of unknown variables explains the fault sensitivity defined in Equation (26). For example, using $yRMC_{1}$ to estimate $q_{CH1}$ makes the $R_{172}$ insensitive to fault $f_{VCH1}$.

## 4. Residual Selection

A large number of residual generator candidates were found in the previous section using structural analysis. However, implementing all candidates is intractable and unnecessary, assuming that only a subset of 10 faults is considered. The high number of residual generator candidates leads to a residual selection problem that is not observed in FDI systems that are not based on structural methods. This problem is addressed in the following to select the most efficient residual generator candidates in terms of fault sensitivity and isolability so as to detect and isolate the predefined faults.

In order to formally build the residual selection problem, some basic model-based diagnosis notions [33] related to fault detection and isolation are recalled. The outputs of a group of residual generators ($R=\left\{R_{1},\cdots ,R_{n_{r}}\right\},n_{r}\in N$) generates a fault signature (*S*) when a fault is injected according to the fault sensitivity of *R*. Only the residuals that were sensitive to this fault deviated from zero.

**Definition** **6** (Fault signature)**.** *For a set of residual generators (R), the fault signature ($S_{f}$) of a fault (f) contains all the residuals ($R_{f}\subseteq R$) sensitive to f.*

Fault isolation is achieved if the set of residual generators (*R*) generates a unique set of fault signatures for each fault:

**Definition** **7** (Fault signature isolability)**.** *A fault (f) is isolable using a set of residual generators (R) if its fault signature ($S_{f}$) is unique when compared to the other fault signatures.*

The objective is to find the minimal subset of residuals capable of detecting and isolating the predefined group of faults. The group of residuals must generate a unique fault signature for each fault. Considering $R_{all}$ as the group of all residual generator candidates, the residual selection problem can be formally defined as the following optimization problem:

(28) $$\begin{array}{cc} \min_{R\subseteq R_{all}}\; & \left|R\right|\\ s.t.\; & S={\left\{S_{1},S_{2},\cdots ,S_{n_{f}}\right\}}_{\neq }\\ \; & S\neq 0 \end{array}$$

where |R| is the cardinality of subset *R* and $n_{f}$ is the number of faults. The same optimization problem is defined was [49]. However, the solution to this problem was not addressed, and the paper’s main goal was to find a subset of residuals that would provide the most unique fault signatures.

A method to solve the optimization problem (28) was first proposed in our previous work [32] but only applied to an illustrative scenario. The proposed residual selection algorithm is applied here to the full rocket engine case study, with ten faults to be detected and isolated.

### Minimal Residual Selection Algorithm

The minimal number of residuals ($n_{min}$) needed to isolate $n_{f}$ faults based on their fault signatures must agree with the following inequality:

(29) $$2^{n_{min}}\geq n_{f}+1.$$

The selection algorithm takes the group of all residual generator candidates ($R_{all}$) and computes all possible subsets of $n_{min}$ elements. Then, the fault-signature isolability of each fault for each subset is tested. The subsets that generate unique fault signatures for each fault are the solution to the optimization problem (28). The number of all possible subsets of $n_{min}$ elements is defined as

(30) $$n_{c}=\frac{n_{R}!}{\left(n_{R}-n_{min}\right)!n_{min}!}$$

where $n_{R}$ is the number of residual generator candidates.

If the number of residual generator candidates ($n_{R}$) is too large, a combinatorial explosion results. To avoid this problem, pre-selection of the residual generator candidates is performed before computing and testing all possible subsets. First, the residuals are separated into two classes based on their fault sensitivity:

- **Detectability class:** For each fault (f∈F), list every residual sensitive to this fault ($R_{df}\in R_{all}$);
- **Undetectability class:** For each fault (f∈F), list every residual that is not sensitive to this fault ($R_{uf}\in R_{all}$).

Subsequently, the most suitable residual generator of each class is selected. The selection criteria are based on the method of residual generation to be employed. Two model-based methods for residual generation are considered here: the sequential residual generation method [50] and observer-based residual generation [51]. The aim is to select the residual generators composed of equations that are suitable for the selected residual generation methods. In addition, the number of equations is taken into account, assuming that each equation has a level of uncertainty. The selection criteria are defined as follows:

1. Choose the residual generators composed of Ordinary Differential Equations (ODEs) or the Differential Algebraic system of Equations (DAE) of index 1.
2. Select the residual generators with minimal “state cardinality”, which means the residuals with a minimal number of differential equations, which are equivalent to the state dimension of the corresponding observer.

The first criterion is to select candidates that can be easily written as ODEs. DAE systems of index 1 can be transformed into ODEs by using the first-order derivative of their algebraic equations [52]. The second criterion selects candidates with minimal cardinality to minimize the level of uncertainty brought about by the equations. The residuals that fit both criteria of each class are then used to search for a solution to the optimization problem (28).

A procedure to find the minimal subset of residuals capable of detecting and isolating a predefined group of faults is described by Algorithm 1. The procedure has three inputs: a list of all residual generators ($R_{all}$), the list of faults (*F*), and the minimal number of residuals ($n_{min}$). The output is a list of subsets of $R_{all}$ with $n_{min}$ elements capable of detecting and isolating all faults in *F*.

The algorithm first computes the detectability and undetectability classes; then, a first loop is used to filter the residuals based on both criteria. If the isolability requirements of the pre-selected group of residuals ($R_{f}$) are not met, the selection criteria are relaxed (rCons). Then, all possible subsets of the pre-selected residuals containing $n_{min}$ elements are computed ($R_{S}$). The isolability of each subset is verified, and only the subsets that can isolate the faults (*F*) are considered the solution ($R_{min}$). The other procedures used in Algorithm 1 are described below.

- The DetectabilityClass(R,F) for each fault (f∈F) lists all residuals from *R* that are sensitive to this fault and returns $n_{f}$ subsets of residuals corresponding to each fault.
- The UndetectabilityClass(R,F) for each fault (f∈F) lists all residuals from *R* that are not sensitive to this fault and returns $n_{f}$ subsets of residuals corresponding to each fault.
- For each detectability class (*d*) and undetectability class (*u*), FilterResiduals(d,u,rCons) filters the residuals considering cardinality and equation structure criteria. If the flag (rCons) is activated, the cardinality criteria are relaxed, and the list of residuals ($R_{f}$) that fit all filtering criteria is returned.
- CheckIsolability(R,F) checks if a group of residuals (*R*) generates unique fault signatures for each fault (f∈F); 1 is returned if true and 0 if false.
- ComputeSubsets($R,n_{min}$) computes all possible combinations of residuals from *R* separated into groups of $n_{min}$ residuals and returns a list containing all possible combinations.

**Algorithm 1:** Residual Selection Algorithm**Inputs**: Set of residual generators $R_{all}$, List of faults *F*, minimum number of residuals $n_{min}$**Output**: Subsets of $R_{all}$ with minimal cardinality $R_{min}$ **procedure**
ResidualSelection(*R*,*F*)  d←detectabilityClass(*R*,*F*)  u←undetectabilityClass(*R*,*F*)  rCons←0  isol←0  **while** isol=0 **do**   $R_{f}\leftarrow$filterResiduals(d,u,rCons)   **if** checkIsolability($R_{f}$,*F*) **then**    isol←1   **else**    rCons←rCons+1  $R_{S}\leftarrow$computeSubsets($R_{f}$,$n_{min}$)  k←0  **for all** $R_{i}\in R_{S}$ **do**   **if** checkIsolability($R_{i},F$) **then**    $R_{min}\left(k\right)\leftarrow R_{i}$    k←k+1   **if** $R_{min}=$ **then**    $n_{min}\leftarrow n_{min}+1$  **return** $R_{min}$


Having the set of residual generators that will be used for fault detection and isolation, the theoretical Fault Signature Matrix (FSM) of the subset can be defined. The FSM binary codifies all fault signatures of a set of faults ($f_{1},f_{2},\cdots f_{n_{f}}$) on a set of residual generators ($R_{1},R_{2},\cdots R_{n_{r}}$) [48]. The FSM has $n_{f}$ columns and $n_{r}$ lines. If a residual generator ($R_{i}$) is sensitive to a fault ($f_{j}$), the *i*-th line cell of the *j*th column is marked 1 and 0 otherwise. Considering that Algorithm 1 guarantees that all fault signatures are unique, each column of the FSM is different.

The residual selection algorithm is illustrated for a simple example. Consider a set of three faults ($F=\{f_{1},f_{2},f_{3}\}$) and three residual generators ($R=\{R_{1},R_{2},R_{3}\}$). The fault sensitivity of each residual generator is defined by the fault signature matrix in Table 2.

Three faults generate six detectability and undetectability classes. Consider that the FilterResiduals( )procedure returns all three residuals ($R_{f}=R$), i.e., all residual generators from *R* are composed of ODEs or DAEs of index 1 and have the same cardinality. The minimal number of residuals required to isolate three faults is $n_{min}\geq 2$. The ComputeSubsets( )function returns three possible subsets of two residuals generators:

(31) $$R_{S}=\left\{R_{1},R_{2}\right\},\left\{R_{2},R_{3}\right\},\left\{R_{1},R_{3}\right\}$$

The CheckIsolability( )procedure confirms that all three subsets expressed in Equation (31) are capable of detecting and isolating the three considered faults ($R_{min}=R_{S}$). It is clear from the fault signature matrix illustrated in Table 2 that all three subsets of Equation (31) generate a unique fault signature for each fault.

## 5. Numerical Residual Sensitivity

**Figure 4:**
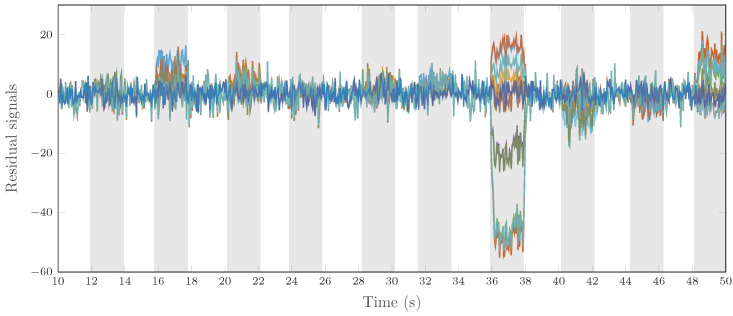
28 different residual signals when faults are injected.

In practice, the residual sensitivity may vary according to various factors, such as the equations and measurements used to compute the residuals. To illustrate the different sensitivities that residuals may have in practice, Figure 4 displays 28 residual signals when 10 different faults are injected. Each fault is active in turn in the gray-shaded areas. The numerical fault sensitivity varies according to the residual generator. For example, when the seventh fault is injected at 36 s, some residuals are much more affected than others.

To achieve the best performance in detecting and isolating faults, it is suitable to implement the subset of residuals with the highest fault sensitivity. A new method to compute the residual numerical fault sensitivity is presented. The method calculates the residuals’ sensitivity in a practical way on a simulated scenario, where the residual signals without faults are compared with the residuals when faults are injected. This comparison involves calculating the signal-to-noise ratio, using the signal without faults as the baseline for noise. Each residual has one residual sensitivity index (RSI) for each fault. The subsets that include residuals with the highest RSIs are then considered the most effective for fault detection and isolation.

The theoretical fault signature matrix contains information about the fault sensitivity of each residual. In order to quantify this sensitivity and compare the different sensitivities between residuals, a Numerical Residual Sensitivity Matrix (NRSM) is constructed. This matrix has the same dimensions as the FSM, but the Boolean values are replaced by an RSI, varying within the range of [0,1]. The RSI is defined as follows:

**Definition** **8**. *Residual Sensitivity Index (RSI): Consider a set of residual signals ($r=\{r_{1},r_{2}\cdots ,r_{n}\}$), all of which are sensitive to one fault ($f_{j}$). Assuming that $r_{i,0}$ is the residual signal when all faults that affect $r_{i}$ are zero and $r_{i,f_{j}}$ is the residual signal when fault $f_{j}$ is injected, the RSI of residual $r_{i}$ concerning fault $f_{j}$ is defined as follows:*

(32) $$RSI_{i,j}=\frac{SNR(r_{i,f_{j}},r_{i,0})}{\max_{k}\left(SNR\left(r_{k,f_{j}},r_{k,0}\right)\right))}$$

*where $SNR(r_{i,f_{j}},r_{i,0})$ is the signal-to-noise ratio between the signal ($r_{i,f_{j}}$) and the noise ($r_{i,0}$):*

(33) $$\begin{array}{c} \begin{array}{cc} \; & SNR\left(r_{i,f_{j}},r_{i,0}\right)=10\log_{10}\left(\frac{P_{signal}\left(r_{i,f_{j}}\right)}{P_{noise}\left(r_{0,f_{j}}\right)}\right)=10\log_{10}{\left(\frac{\sqrt{\frac{1}{n_{s}}}\sum_{l=1}^{n_{s}}r_{i,0,l}^{2}}{\sqrt{\frac{1}{n_{s}}}\sum_{l=1}^{n_{s}}r_{i,f_{j},l}^{2}}\right)}^{2} \end{array} \end{array}$$

*where $n_{s}$ is the number of samples.*

In other words, the RSI of residual signal $r_{i}$ of fault $f_{j}$, denoted as $RSI_{i,j}$, is a max normalization of the signal-to-noise ratio considering all residual signals that are sensitive to fault $f_{j}$. For instance, consider two residual signals ($r_{i}$ and $r_{k}$), both of which are sensitive to fault $f_{j}$. If $RSI_{i,j}=2RSI_{k,j}$, then signal-to-noise ratio of residual $r_{i}$ is two times greater than the signal-to-noise ratio of residual $r_{k}$ when $f_{j}$ is injected. The number of RSIs that can be computed from a residual depends on the sensitivity of the residual. If residual $r_{i}$ is sensitive to three faults, for example ($f_{1},f_{2}$, and and $f_{3}$), three RSIs can be computed ($RSI_{i,1}$, $RSI_{i,2}$, and $RSI_{i,3}$).

The numerical residual sensitivity matrix is constructed by calculating the RSI between each residual and fault (Equation (32)) and defining $NRSM_{i,j}=RSI_{i,j}$. Two other indices can be calculated from the RSI: the Overall Residual Sensitivity Index (ORSI) and the Subset Sensitivity Index (SSI).

**Definition** **9**. *Overall Residual Sensitivity Index (ORSI): Consider a residual signal ($r_{i}$) sensitive to a group of faults ($f=\{f_{1},f_{2},\cdots ,f_{nf}\}$); the ORSI is the geometric mean of all RSIs that can be calculated from $r_{i}$:*

(34) $$ORSI_{i}={\left(\prod_{j=1}^{n_{f}}RSI_{i,j}\right)}^{\frac{1}{nf}}$$

Each residual has only one ORSI. From the ORSI, the SSI is obtained:

**Definition** **10**. *Subset Sensitivity Index (SSI): Consider a subset (S) composed of $r=\{r_{1},r_{2},\cdots ,r_{nr}\}$ residual. The $SSI_{S}$ of subset S is defined as follows:*

(35) $$SSI_{S}={\left(\prod_{i=1}^{n_{r}}ORSI_{i}\right)}^{\frac{1}{nr}}$$

The definition of ORSI and SSI using the geometric mean may lead to SSI values equal to zero if one residual does not present in the same theoretical sensitivity in simulation. If residual $r_{i}$ is, in theory, sensitive to $f_{j}$ but the same sensitivity is not observed in simulation ($RSI_{i,j}=0$), it follows that $ORSI_{i}=0$ and that the SSI of all subsets that use $r_{i}$ will also be zero.

The reduced-order example from Table 2 is now used to illustrate how the RSI, ORSI, and SSI are calculated. Consider that three residual generators ($R=\{R_{1},R_{2},R_{3}\}$) generate three different residuals in the fault-free scenario ($r_{0}=\left\{r_{1,0},r_{2,0}.r_{3,0}\right\}$). In addition, three different residuals are generated when each fault is injected ($r_{fk}=\left\{r_{1,fk},r_{2,fk},r_{3,fk}\right\}$, k∈[1,3]). Consider that when $f_{y_{2}}$ is injected, $r_{2}$ and $r_{3}$ have the same SNR. When $f_{y_{1}}$ is injected, $r_{1}$ has an SNR ten times greater than that of $r_{2}$, and when $f_{u}$ is injected, $r_{1}$ has an SNR two times greater than that of $r_{3}$. From (32), the following RSI is obtained, which yields the NRSM defined in Table 3:

(36) $$\begin{array}{c} \begin{array}{cc} \; & RSI_{1,1}=RSI_{1,2}=RSI_{2,1}=RSI_{3,1}=1\;RSI_{2,2}=0.1\;RSI_{3,3}=0.5 \end{array} \end{array}$$

From (36), the ORSI of each residual generator can be calculated:

(37) $$ORSI_{1}=1\;ORSI_{2}=\sqrt{0.1}\;ORSI_{3}=\sqrt{0.5}$$

The results from the residual selection problem show that any combination of two residuals from Table 3 is sufficient to detect and isolate the three faults. By using the ORSI obtained in (37), the following SSIs are derived:

(38) $$SSI_{1}=\sqrt{0.1}\approx 0.36\;SSI_{2}=\sqrt{0.05}\approx 0.22\;SSI_{3}=\sqrt{0.5}\approx 0.71$$

where $SSI_{1}=\left\{r_{1},r_{2}\right\}$, $SSI_{2}=\left\{r_{2},r_{3}\right\}$, and $SSI_{3}=\left\{r_{1},r_{3}\right\}$. In (38), subset $SSI_{3}$ is the highest SSI and, therefore, achieves the best performance in detecting and isolating faults.

## 6. Fault Isolation

Fault isolation is performed by comparing all possible fault signatures with the observed fault signature from the residual analysis. Considering that each fault signature is unique, a fault is isolated if a perfect match is observed.

### Fault Signature Analysis

If a perfect fault signature match is not observed but a fault is detected, the goal is to predict the most probable fault using the information from the NRSM defined in Section 5.

To achieve this, an isolation fault index (Is) is calculated. The faults with the highest isolation indices are considered the most likely to be active in the system. The concepts of a numerical fault signature (Nfs) and an observed fault signature (Ofs) are introduced here. An Nfs is equivalent to a theoretical fault signature (Tfs) but obtained from the NRSM instead of the FSM. An Ofs is obtained by analyzing the residual signal and deciding which residuals deviated from fault-free behavior [51].

The isolation index (Is) for a given fault (*f*), with a theoretical fault signature (Tfs) and numerical fault signature (Nfs) for an observed fault signature (Ofs), can be calculated in two main steps:

1. Rewarding when the residuals behave as expected, where the theoretical fault signature (Tfs) matches the observed fault signature Ofs.
2. Penalizing when the residuals do not behave as expected. In this scenario, either the theoretical fault signature (Tfs) expected a residual to be sensitive but it was not, which is associated with the probability of non-detection, or the Tfs expected a residual not to be sensitive but it was, which is associated with the probability of a false alarm.

The isolation index (Is) is determined by aggregating all rewards and penalties. The crucial concept is incorporated in step 2, where a penalty associated with the probability of non-detection is computed. This penalty is derived from the numerical fault signature (Nfs) of the residual. For example, a residual with higher fault sensitivity will incur a larger penalty than one with lower fault sensitivity. The formal description of the procedure to calculate the isolation index (Is) is presented in Algorithm 2.
**Algorithm 2:** Isolation index of a fault (*f*) algorithm**Inputs**: Theoretical fault signature Tfs, observed fault signature Ofs, numerical fault signature Nfs**Output**: Isolation index Is **procedure** IsolationIndex(Tfs,Nfs,Ofs)  Cfs←Xnor(Tfs,Ofs)  Is←0  nr←Length(Tfs)  **for all** i∈nr **do**   **if** $Cfs_{i}=1$ and $Ofs_{i}=1$ **then**    Is←Is+1   **if** $Cfs_{i}=1$ and $Ofs_{i}=0$ **then**    Is←Is+1   **if** $Cfs_{i}=0$ and $Ofs_{i}=0$ **then**    Is←Is−1   **if** $Cfs_{i}=0$ and $Ofs_{i}=1$ **then**    $Is\leftarrow Is-Nfs_{i}$  **return** Is


To illustrate how Algorithm 2 works, let us apply it to the example from Section 5. Consider the numerical residual sensitivity matrix defined in Table 3, where each line corresponds to a numerical fault signature and the following observed fault signature: Ofs={1,0,0}. Consider that all three residuals are used to isolate the fault.

The observed fault signature does not match with any theoretical fault signature. Applying Algorithm 2 for each fault, the following isolation index is obtained:

(39) $$Is_{f1}=-1-1-1=-3\;Is_{f2}=1-0.1+1=1.9\;Is_{f3}=1+1-0.5=1.5,$$

which indicates $f_{2}$ as the active fault in the system. The theoretical signatures from faults $f_{2}$ and $f_{3}$ have the same “distance” from the observed fault signature (Ofs), with two perfect matches from two residuals. Fault $f_{2}$ has a greater fault isolation index because residual $r_{2}$ is less sensitive than residual $r_{3}$, so it is more likely that $r_{2}$ did not detect $f_{2}$ than $r_{3}$ did not detect $f_{3}$.

## 7. Application to the Multi-Engine Cluster

The multi-engine cluster model presented in Section 2 was implemented in ${\mathrm{Simulink}}^{®}$. Experimental results are not yet possible, as reusable launch vehicles are still under development in Europe [53].

To replicate the behavior of the cluster when a fault is injected, a MIMO control system with three PIDs for each engine was designed. The control system is designed to keep each engine around a nominal operating point of 1000 kN Each PID uses a classical configuration [40] where three outputs are controlled by three inputs. The outputs are the mixture ratio in the combustion chamber and gas generator chamber and the combustion chamber pressure ($y_{PID,i}={\left[RMC_{i},RMG_{i},P_{Ci}\right]}^{T}$). The control inputs are the directional valves and the gas generator ${LH}_{2}$ and LOX valves ($u_{PID,i}={\left[Zr_{i},VG_{Hi},VG_{Oi}\right]}^{T}$), where the index $._{i}$ denotes the i-th engine. The control system is configured to have a settling time to the step response of 2 s without overshooting.

### 7.1. Measurement Noise and Sampling Frequency

Measurement noise consistent with realistic measurement assumptions is added to the simulation. The noise information is presented in Table 4. The sensors are configured to measure at a frequency of 10 kHz

### 7.2. Residual Selection for the Multi-Engine Cluster

The structural analysis from Section 3 returned 16,441 residual generator candidates to detect and isolate a group of 10 faults. The residual selection algorithm presented in Section 4 was applied to find the subsets of residuals capable of isolating all faults. This led to the extraction of 20 detectability and undetectability classes from ten faults ($n_{f}=10$). The first pre-selection of residual generator candidates performed by the filterResiduals function returned 28 different residual generator candidates ($n_{r}=28$) after the analysis of 16,441 candidates. These 28 selected candidates can isolate all faults. According to Equation (30), at least 4 residuals are necessary to isolate 10 faults ($n_{min}\geq 4$).

The residual selection Algorithm 1 was run two times to investigate if the size of the subsets influences fault detection and isolation performance. In the first run, the minimal number of residuals was defined as $n_{min}=4$. There were 23,741 subsets of four residuals, and 6 subsets met the detectability and isolability requirements ($dim(R_{min,4})=6$). The second time, the minimum number of residuals was defined as $n_{min}=5$. There were 118,755 subsets of five residuals, and 594 subsets met the detectability and isolability requirements ($dim(R_{min,5})=594$).

The results obtained from the selection Algorithm 1 can be compared with the selection algorithm presented in [33], where a more restrictive isolability constraint is used. When applying the selection algorithm from [33] on the 28 residuals, the algorithm did not find any solution capable of isolating all faults. The algorithm requires that 90 constraints be respected in order to isolate the same 10 faults. The 28 residuals were not able to respect 3 out of 90 constraints.

To illustrate some results returned by the residual selection algorithm, three subsets of residual generators that theoretically can detect and isolate all faults are defined below.

(40) $$\begin{array}{c} \begin{array}{cc} \; & Sb4_{3}=\left[R_{172},R_{716},R_{720},R_{1004}\right]\\ \; & Sb5_{223}=\left[R_{172},R_{186},R_{710},R_{716},R_{1000}\right]\\ \; & Sb5_{263}=\left[R_{172},R_{186},R_{716},R_{720},R_{1000}\right]. \end{array} \end{array}$$

**Figure 5:**
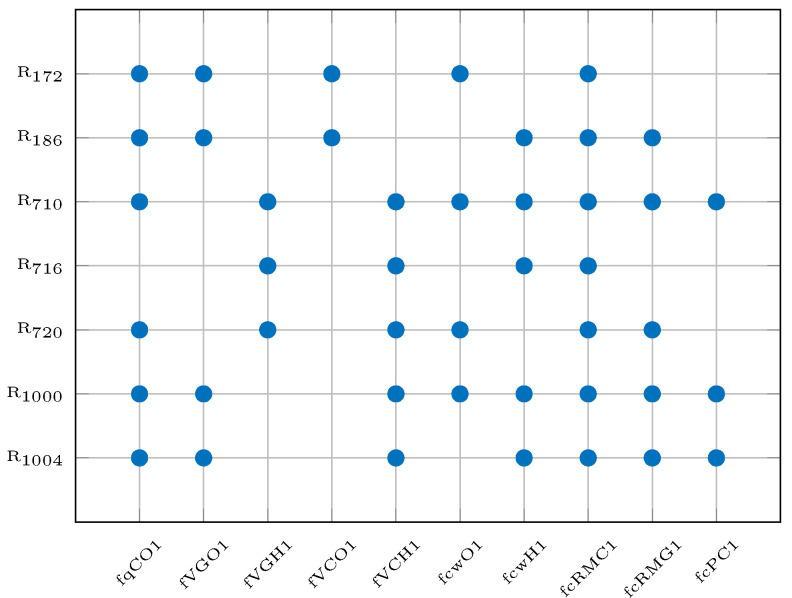
Fault signature matrix.

The fault signature matrix of the residual generators used by the subsets from (40) are illustrated in Figure 5. It is possible to see that all three subsets generate a unique fault signature for each fault.

### 7.3. Residual Generation

The sequential residual generation method [50] is used for residual generation. It is a fundamental method for residual generation that relies on the model equations to verify the consistency between them and the known variables. The process of calculating the residual is described in [54]. Let $V_{1}\subseteq V$ be the subset of equations of a residual generator ($R_{1}$) with unknown variables ($X_{1}\subseteq X$). By choosing one equation from $R_{1}$ to check the consistency ($v_{i}\in V_{1}$), the remaining subset of equations, which initially had a degree of redundancy equal to one, now forms $V_{1}^{1}=V_{1}\setminus \left\{v_{i}\right\}$ as a just-determined set of equations. The generation method first solves the just-determined problem to estimate all unknown variables ($X_{1}$). Then, the consistency equation ($v_{i}$) is used to generate a residual. The only configuration needed to implement this method is the definition of the equation that will be used to check the consistency ($v_{i}$). This generation method is automated by the fault diagnosis toolbox [28].

**Figure 6:**
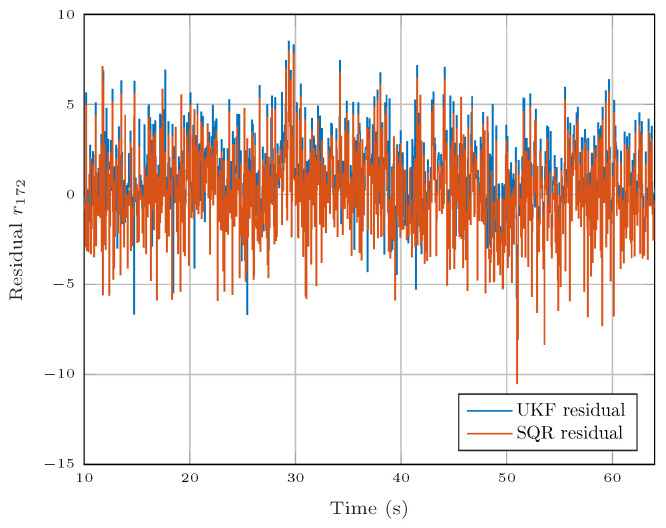
Residual $r_{172}$ generated by two different methods.

Another residual generation technique is based on state observers [51]. The main advantage of state observers over the sequential residual generation method is the use of measurements (*y*) to adjust the state estimation ($\hat{x}$), which makes it more robust to model uncertainty and unmodeled dynamics. Under some specific constraints, the residuals generated by the two methods have a very similar form. Figure 6 illustrates the signal generated by residual $R_{172}$ (24) using the sequential residual generation method and a method based on the Unscented Kalman Filter (UKF) state observer [55], where the UKF process noise covariance matrix (*Q*) is defined as one hundred times smaller than the measurement noise covariance matrix (*R*).

The rest of the analysis is performed using the residuals generated by the sequential residual generation method due to its simplicity of implementation. However, the results could be extended to observer-based residual generation as illustrated in Figure 6.

### 7.4. RSI, ORSI, and SSI Computation

To compute the SSI for each of the 600 subsets, it is necessary to compute the RSI and ORSI of the 28 residual generators selected in Section 7.2. To illustrate how those indices can be calculated, two residual generators are used as examples: $R_{710}$ and $R_{720}$. The sensitivity of those residuals is defined in Figure 5. Residual generators $R_{710}$ and $R_{720}$ are sensitive to eight and six faults, respectively, which leads to eight RSI values for $R_{710}$ and six RSI values for $R_{720}$.

**Figure 7:**
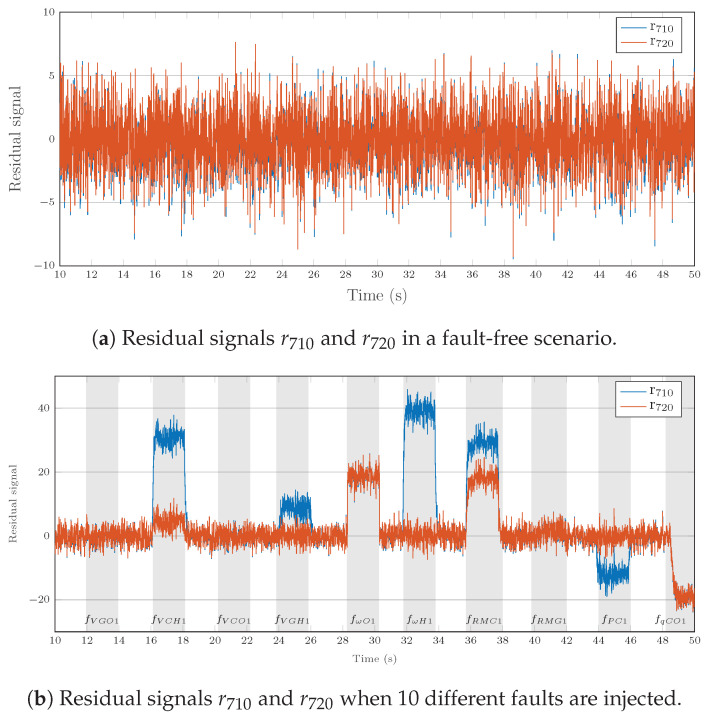
Residual signals $r_{710}$ and $r_{720}$ in two different scenarios.

The first step is to use the residual generator (*R*) to generate a residual signal (*r*). The sequential residual generation method is used with the aid of the fault diagnosis toolbox [28]. The residual signal in a fault-free condition generated by both residual generators is depicted in Figure 7a. The residual signal when all ten considered faults are injected is illustrated in Figure 7b, where the faults are active in the gray-shaded areas.

Using Equation (33) to compute the signal-to-noise ratio between the noise signal depicted in Figure 7a and the signal from Figure 7b, the values presented in Table 5 are obtained. Based on the signal-to-noise ratio, the residual signal of $r_{710}$ is more sensitive than $r_{720}$ considering faults $f_{VCH1}$, $f_{VGH1}$, and $f_{RMC1}$, while residual $r_{720}$ is more sensitive than $r_{710}$ to faults $f_{RMG1}$ and $f_{qCO1}$. The largest difference is concerning fault $f_{VGH1}$, where $r_{710}$ has a signal-to-noise ratio 54 times higher than that of $r_{720}$.

In order to obtain the RSI values, the signal-to-noise ratio must be normalized by dividing the signal-to-noise ratio value by the maximum value considering all 28 residual signals. Repeating the process of generating residual signals using the SQR and computing the signal-to-noise ratio of each residual for the remaining 26 residual generators and performing the max normalization, the RSI values for the used residuals form the subsets defined in Equation (40) in Table 6.

From (34), the ORSI is obtained by computing the geometric mean of the RSI values for each residual signal. For $r_{710}$ and $r_{720}$, using the RSI values defined in Table 6, the following ORSIs are obtained:

(41) $$\begin{array}{c} ORSI_{r_{710}}={\left(1\;\cdot \;1\;\cdot \;0.91\;\cdot \;1\;\cdot \;0.76\;\cdot \;0.03\;\cdot \;1\;\cdot \;0.76\right)}^{\frac{1}{8}}=0.58 \end{array}$$

(42) $$\begin{array}{c} ORSI_{r_{720}}={\left(0.29\;\cdot \;0.02\;\cdot \;0.91\;\cdot \;0.61\;\cdot \;0.04\;\cdot \;0.76\right)}^{\frac{1}{6}}=0.21 \end{array}$$

Having defined the ORSI for all residuals, the SSI of the subsets can be defined. The SSI of subsets $Sb5_{223}$ and $Sb5_{263}$, for example, is computed by taking the geometric mean of the ORSI values:

(43) $$\begin{array}{cc} \; & SSI_{Sb5_{223}}={\left(ORSI_{r_{172}}\;\cdot \;ORSI_{r_{186}}\;\cdot \;ORSI_{r_{716}}\;\cdot \;ORSI_{r_{1000}}\;\cdot \;ORSI_{r_{710}}\right)}^{\frac{1}{5}}=0.54 \end{array}$$

(44) $$\begin{array}{cc} \; & SSI_{Sb5_{263}}={\left(ORSI_{r_{172}}\;\cdot \;ORSI_{r_{186}}\;\cdot \;ORSI_{r_{716}}\;\cdot \;ORSI_{r_{1000}}\;\cdot \;ORSI_{r_{720}}\right)}^{\frac{1}{5}}=0.43 \end{array}$$

From (44), we can infer that $SSI_{Sb5_{223}}>SSI_{Sb5_{263}}$, which indicates that subset $Sb5_{223}$ achieves better FDI performance than $Sb5_{263}$. In addition, subset $Sb5_{223}$ has the highest SSI among the 594 subsets of five residuals.

For the SSI values of the other subsets, the six subsets of four residuals have a mean SSI of 0.65, and the 594 subsets of five residuals have a mean SSI of 0.28. The subset sensitivity cannot be compared between the two families of subsets (with four and five residuals) because it uses a different value to normalize the RSI. The precise value of each subset sensitivity index is analyzed using Monte Carlo simulation results in Section 7.6.

### 7.5. Residual Evaluation

The residual evaluation was carried out by applying Wald’s Sequential Probability Ratio Test (SPRT) [56] to detect a change in mean μ for a given residual (*r*). The SPRT is based on a variable horizon; if a decision cannot be taken for a given risk, the decision is reported, and another sample is used. Usually, SPRT detects faults faster than classical ratio tests with fixed observation horizons.

The SPRT equation used to detect any mean change is defined in [57]:

(45) $$\begin{array}{cc} \; & \frac{\sigma_{0}^{2}}{\mu_{f}}\ln A+\frac{k\mu_{f}}{2}\underset{H_{0}}{<}\left|\sum_{i=1}^{k}r_{i}-\mu_{0}\right|\underset{H_{1}}{<}\frac{\sigma_{0}^{2}}{\mu_{f}}\ln B+\frac{k\mu_{f}}{2} \end{array}$$

where $\sigma_{0}^{2}$ and $\mu_{0}$ are the nominal variance and mean of the residual *r*, respectively; $\mu_{f}$ is the mean threshold; *k* is the number of samples; and constants *A* and *B* are configured according to the probability of false alarm ($P_{f}$) and non detection ($P_{nd}$), respectively:

(46) $$A=\frac{P_{nd}}{1-P_{f}}\;B=\frac{1-P_{nd}}{P_{f}}.$$

The test (45) has two outputs: $H_{0}$ if residual *r* has a nominal mean of $\mu_{0}$ or $H_{1}$ if the residual *r* has a absolute mean greater than $\mu_{f}$ considering the risk defined by *A* and *B*.

The sequential probability ratio test was implemented for each residual generator. To configure the test, a fault-free simulation was run for 300 s, and the expected mean ($\mu_{0}$) and standard deviation ($\sigma_{0}$) of each residual in a fault-free condition were computed. The hypothesis test is configured to detect a fault if the residual mean ($\mu_{f}$) exceeds the expected mean by more than 1.8 times the expected standard deviation:

(47) $$\mu_{f}=\mu_{0}+1.8\sigma_{0}.$$

To avoid false alarms, the SPRT is configured to take a decision after 100 measurements, which corresponds to minimum detection time of 0.1 s The probability of non-detection and the false-alarm were set at $P_{nd}=0.05$ and $P_{f}=0.01$, respectively.

### 7.6. Monte Carlo Simulations

To analyze the performance of the selected subsets in detecting and isolating the predefined faults and the relation between the subset sensitivity index and its detection and isolation rates, 500 Monte Carlo simulations were carried out. All ten faults were injected during the simulation remained active for 2 s after injection. The fault injection time and magnitude varied according to a predefined range shown in Table 7. All fault magnitudes are expressed in terms of the percentage of their corresponding value when the multi-engine cluster is at its nominal operating point, i.e., $f_{VGO1}$ represents a blocking that varies from 8.8% to 4% of the nominal value of $VG_{O1}$. The range of magnitudes was intentionally selected to test the sensitivity of the FDI systems with small values.

**Figure 8:**
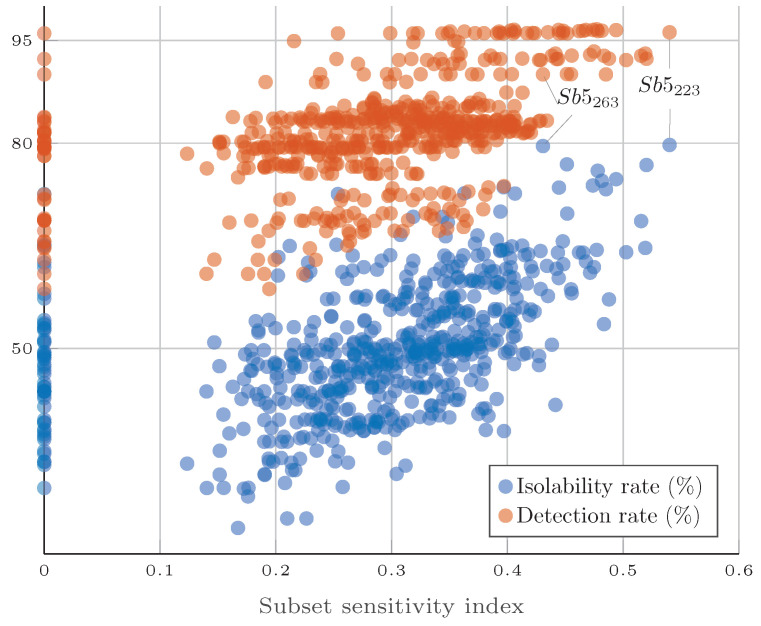
Simulation results: subsets with 5 residuals.

The results obtained with the six subsets of four residual generators are presented in Table 8. For the 595 subsets of five residuals, the simulation results are illustrated in Figure 8, where the detectability and isolability rate of each subset is represented by a circle.

The subsets with four residuals all had a similar sensitivity index, with detection and isolability rates varying within a narrow range. The sensitivity index appears to be more closely correlated with the isolability rate than the detection rate. Due to the limited number of subsets and the small difference between subsets, it is not possible to draw definitive conclusions.

The most interesting results were obtained with the subsets composed of the five residuals shown in Figure 8. The sensitivity index of the subsets varies within a large range, from 0 to 0.5401. From a detection rate perspective, the sensitivity index seems to have a small correlation. Where one sort of orange line is formed, some residual generators are much more sensitive to faults, and if these residuals are part of the subset, the subset present a high detection rate, regardless of the subset sensitivity index. However, a strong correlation can be noticed between the sensitivity index and the isolability rate. It is clear that if a subset has a high sensitivity index, the isolability rate is also high.

The results from Figure 8 also show the utility of the fault signature analysis discussed in Section 6. Subsets with a sensitivity index equal to zero mean that one residual of the subset does not present, in practice, the expected theoretical fault sensitivity. The residual selection problems consider only the theoretical fault sensitivity, so it is not guaranteed that a subset with a sensitivity index of zero can isolate all faults. The strategy of taking into account the residual sensitivity to predict an active fault in the system explains subsets with a zero sensitivity index and non-negligible isolability rates.

The subsets that presented the highest isolability rates are $Sb5_{223}$ and $Sb5_{263}$, with 79.66% and 79.72%, respectively. According to (43) and (5), $Sb5_{223}$ had a higher SSI than $Sb5_{263}$, but in simulation, the performance appears to be similar. The difference between the two subsets is clear when the performance is analyzed in more detail. Subset $Sb5_{223}$ has a higher sensitivity index because its performance in isolating the ten faults is more even when compared with subset $Sb5_{263}$, which is more effective in isolating specific faults. The performance of both subsets in isolating each fault is presented in Table 9.

**Figure 9:**
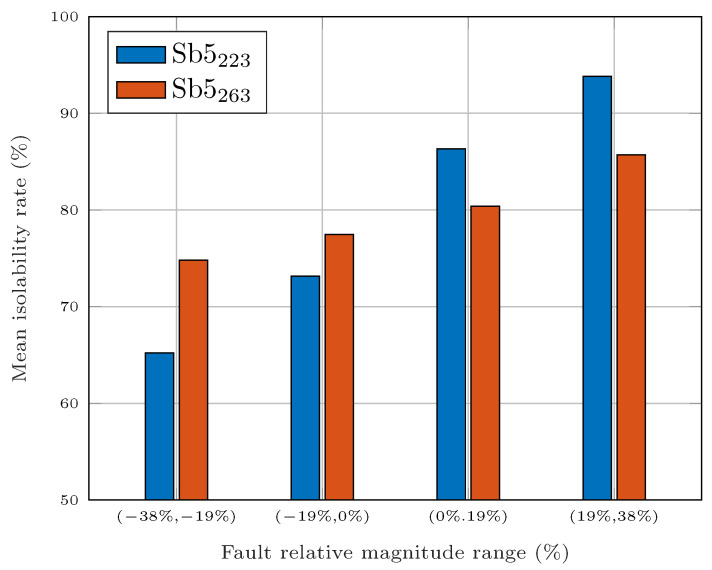
Comparison of $Sb5_{223}$ and $Sb5_{263}$ for different fault magnitudes.

The difference between subsets $Sb5_{223}$ and $Sb5_{263}$ is more prominent when the fault magnitude is taken into account, as illustrated in Figure 9, where the mean isolability rate of all faults is separated by different groups of fault magnitudes. For small fault magnitudes, the isolability rate of $Sb5_{263}$ is superior. However, when the magnitude of the faults is increased, the isolability performance of $Sb5_{223}$ overtakes that of $Sb5_{263}$.

**Figure 10:**
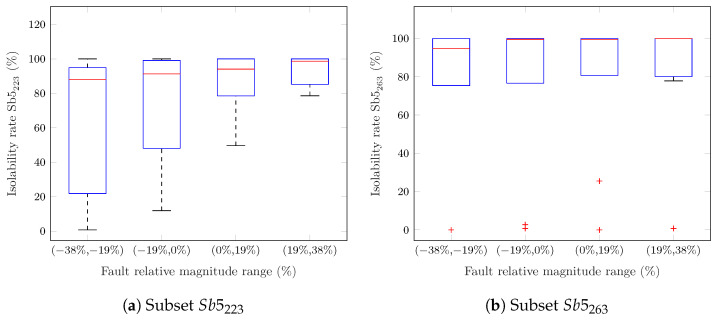
Box-and-whisker diagram.

The spread of the isolability rates obtained for all ten faults separated by fault magnitude is illustrated by the box-and-whisker diagram in Figure 10. It is possible to see that for subset $Sb5_{223}$, the isolability performance increases for all faults when the magnitude is more significant. On the other hand, for subset $Sb5_{263}$, global isolability performance stays similar for all fault magnitudes. In addition, the performance of $Sb5_{263}$ has outliers with an isolability rate close to 0%, even for the highest fault magnitudes, which corresponds to the isolability of fault $f_{VGH1}$ defined in Table 9. The difference between the two subsets is clear, and the choice of the best subset depends on the FDI constraints—for instance, if it is preferable to detect all faults, even with a low isolability rate for small magnitudes ($Sb5_{223}$), or if the global isolability performance should be higher for all fault magnitudes, with some faults not being isolated ($Sb5_{263}$).

## 8. Conclusions

A model-based fault detection and isolation system was developed for ten different actuator and sensor faults for a multi-engine cluster. A model of a multi-engine cluster with three engines was presented; it includes the dynamics of the liquid oxygen tank, feeding lines, and liquid-propellant rocket engines. For the first time, structural analysis was used in a complete multi-engine cluster model. The analysis computed 16,441 independent subsystems that could be used as residual generators for FDI purposes. A residual selection algorithm was proposed to find subsets of candidates capable of detecting and isolating all faults with minimal carnality. The selection algorithm returned 6 subsets of 4 residuals and 594 subsets of 5 residuals theoretically capable of detecting and isolating the faults.

In order to find the subsets with the highest fault sensitivity, a subset sensitivity index (SSI) is introduced. The SSI is calculated based on the residual signal. Two residual generation methods were considered: the sequential residual generation method and an unscented Kalman filter (UKF). The computation of the SSI relies on the residuals generated by the sequential method. The subsets with the highest SSI are, in theory, more sensitive to the faults. The similarities between the residual signals generated by the two methods indicated that the RSI is also valid for residuals generated using the estimations of the UKF. Fault detection was performed by analyzing the residual signal using the sequential probability ratio test for the mean. Fault isolation is performed in two steps. First, the fault is isolated if a perfect match between a theoretical and an observed fault signature is detected. Otherwise, if the observed fault signature does not match any theoretical fault signature, an algorithm that uses information about the numerical residual sensitivity predicts which is the most probable fault to be present in the system.

Monte Carlo simulations were performed under realistic measurement noise assumptions. They showed a high correlation between the SSI and the isolability rate, indicating that the SSI can be used to find the subset that yields the highest FDI performance. The best subsets achieved a detection rate of more than 95% and an isolability rate of 79%.

## 9. Perspectives

The effects of model uncertainty and disturbances could be taken into account. Future work could also consider the form of the fault signature to avoid incorrect isolation of faults. For example, the selection algorithm could search for subsets that produce fault signatures that can be easily distinguished from the others instead of searching for subsets with minimal cardinality.

In addition, in order to restrict the number of residual generator candidates, constraints from the residual generation method could be considered when searching for candidates. For instance, if state observers are used for residual generation, the observability Gramian of the residual generator candidates could be taken into account to find candidates for which an asymptotic observer can be derived.

## Figures and Tables

**Table 1:** Fault information.

Fault	Definition
$f_{qCO}$	CC LOX leakage
$f_{VCH}$	$V_{CH}$ blockage
$f_{VGO}$	$V_{GO}$ blockage
$f_{VCO}$	$V_{CO}$ blockage
$f_{VGH}$	$V_{GH}$ blockage
$f_{PC}$	Bias on measurement of $P_{C}$
$f_{RMC}$	Bias on measurement of RMC
$f_{RMG}$	Bias on measurement of RMG
$f_{\omega H}$	Bias on measurement of ωH
$f_{\omega O}$	Bias on measurement of ωO

**Table 2:** Fault signature matrix (FSM).

	$f_{1}$	$f_{2}$	$f_{3}$
** $R_{1}$ **	0	1	1
** $R_{2}$ **	1	1	0
** $R_{3}$ **	1	0	1

**Table 3:** Numerical residual sensitivity matrix of the reduced-order example.

	$r_{1}$	$r_{2}$	$r_{3}$
** $f_{y_{2}}$ **	0	1	1
** $f_{y_{1}}$ **	1	0.1	0
** $f_{u}$ **	1	0	0.5

**Table 4:** Measurement noise information.

Measurement	Standard Deviation σ (% of the Nominal Value)
$P_{ull},P_{s1},P_{H}$	0.23%
$P_{G},P_{C}$	0.33%
$\omega_{O},\omega_{H}$	0.14%
$RM_{C},RM_{G}$	1%

**Table 5:** Signal-to-noise ratio values for $r_{710}$ and $r_{720}$ (in dB).

	$f_{\mathrm{VGO}1}$	$f_{\mathrm{VCH}1}$	$f_{\mathrm{VCO}1}$	$f_{\mathrm{VGH}1}$	$f_{\mathrm{wO}1}$	$f_{\mathrm{wH}1}$	$f_{\mathrm{RMC}1}$	$f_{\mathrm{RMG}1}$	$f_{\mathrm{PC}1}$	$f_{\mathrm{qCO}1}$
$r_{710}$		22.11		10.92	17.9	23.67	20.23	0.4305	12.79	14.44
$r_{720}$		6.406		0.2175	17.9		16.09	0.6247		14.47

**Table 6:** Numerical residual sensitivity matrix.

	$f_{\mathrm{qCO}1}$	$f_{\mathrm{VGO}1}$	$f_{\mathrm{VGH}1}$	$f_{\mathrm{VCO}1}$	$f_{\mathrm{VCH}1}$	$f_{\omega_{O1}}$	$f_{\omega_{H1}}$	$f_{\mathrm{RMC}1}$	$f_{\mathrm{RMG}1}$	$f_{\mathrm{PC}1}$
$R_{172}$	0.23	0.04	0	0.43	0	0.92	0	0.03	0	0
$R_{186}$	0.87	0.64	0	0.65	0	0	0.89	0.99	0.66	0
$R_{710}$	0.76	0	1	0	1	0.91	1	0.76	0.03	1
$R_{716}$	0	0	0.78	0	0.51	0	0.89	0.69	0	0
$R_{720}$	0.76	0	0.02	0	0.29	0.92	0	0.61	0.04	0
$R_{1000}$	0.75	1	0	0	0.99	0.91	0.99	0.75	1	0.92
$R_{1004}$	0.01	0.41	0	0	0.76	0	0.89	0.02	0.48	0.29

**Table 7:** Fault configuration.

Fault	Time (s)	Magnitude (% of the Nominal Value)
$f_{VGO1}$	12±0.5s	6.4±38%
$f_{VCH1}$	16±0.5s	2.4±38%
$f_{VCO1}$	20±0.5s	0.8±38%
$f_{VGH1}$	24±0.5s	2.0±38%
$f_{\omega O1}$	28±0.5s	0.08±38%
$f_{\omega H1}$	32±0.5s	0.06±38%
$f_{RMC1}$	36±0.5s	4.0±38%
$f_{RMG1}$	40±0.5s	25.5±38%
$f_{PC1}$	44±0.5s	0.08±38%
$f_{fCO1}$	48±0.5s	0.8±38%

**Table 8:** Simulation results: subsets with 4 residuals.

Subset	Detection Rate	Isolability Rate	Sensivity Index (SSI)
$Sb4_{1}$	75.68%	53.18%	0.6758
$Sb4_{2}$	67.38%	50.60%	0.7015
$Sb4_{3}$	76.80%	39.14%	0.5876
$Sb4_{4}$	69.00%	43.76%	0.6490
$Sb4_{5}$	83.78%	43.80%	0.6407
$Sb4_{6}$	81.62%	46.36%	0.6814

**Table 9:** Fault isolation performance.

Fault	Isolability Rate $\mathrm{Sb}5_{223}$	Isolability Rate $\mathrm{Sb}5_{263}$
$f_{VGO1}$	90.00%	100.00%
$f_{VCH1}$	97.60%	97.60%
$f_{VCO1}$	95.40%	97.40%
$f_{VGH1}$	61.60%	0.4%
$f_{\omega O1}$	89.40%	94.00%
$f_{\omega H1}$	99.80%	99.80%
$f_{RMC1}$	43.60%	100.00%
$f_{RMG1}$	39.20%	28.00%
$f_{PC1}$	99.60%	99.60%
$f_{fCO1}$	89.40%	89.40%

## Data Availability

Data is contained within the article.

## Nomenclature

**Acronyms**	
CC	Combustion Chamber
GG	Gas-Generator
${LH}_{2}$	Liquid Hydrogen
LOX	Liquid Oxygen
**Faults**	
$f_{PC}$	Bias on measurement of $P_{C}$ [Pa]
$f_{qCO}$	CC LOX leakage [kg s^−1^]
$f_{RMC}$	Bias on measurement of RMC
$f_{RMG}$	Bias on measurement of RMG
$f_{VCH}$	$V_{CH}$ valve blockage [$m^{2}$]
$f_{VCO}$	$V_{CO}$ valve blockage [$m^{2}$]
$f_{VGH}$	$V_{GH}$ valve blockage [$m^{2}$]
$f_{VGO}$	$V_{GO}$ valve blockage [$m^{2}$]
$f_{wH}$	Bias on measurement of $\omega_{H}$ [${\mathrm{rad}\;s}^{-1}$]
$f_{wO}$	Bias on measurement of $\omega_{O}$ [${\mathrm{rad}\;s}^{-1}$]
**Inputs**	
$V_{CH}$	CC LH_2_ valve surface [$m^{2}$]
$V_{CO}$	CC LOX valve surface [$m^{2}$]
$V_{GH}$	GG LH_2_ valve surface [$m^{2}$]
$V_{GO}$	GG LOX valve surface [$m^{2}$]
$Z_{r}$	Hot gas valve resistance [${kg}^{-1}m^{-1}$]
**Known parameters**	
α	Sound speed [${ms}^{-1}$]
ρ	Density [${\mathrm{kg}m}^{-3}$]
ac,bc,cc	Pump torque polynomial coefficients
ap,bp,cp	Pump pressure polynomial coefficients
*D*	Pipe diameter [m]
$f_{r}$	Pipe friction factor
$I_{G}$	GG fluid inertia [$kg\;\times \;m^{2}$]
$I_{T}$	Turbine fluid inertia [$kg\;\times \;m^{2}$]
*J*	Turbopump inertia [$kg\;\times \;m^{2}$]
$k1_{C},k2_{C}$	CC coefficients
$k1_{T},k2_{T}$	Turbine coefficients
$k_{yTH}$	Hot gas ${\mathrm{LH}}_{2}$ line resistance [${kg}^{-1}m^{-1}$]
*L*	Pipe length [m]
$R_{C}$	CC line resistive coefficient [${mkg}^{-1}$]
$R_{G}$	GG line resistive coefficient [${mkg}^{-1}$]
$R_{CG}$	Common line resistive coefficient [${mkg}^{-1}$]
Re	Reynolds number
*S*	Pipe surface area [$m^{2}$]
$T_{C}$	CC inner temperature [K]
$T_{G}$	GG inner temperature [K]
*V*	Pipe volume [$m^{3}$]
**Subscript**	
$._{C}$	Subscript for Combustion Chamber (CC)
$._{G}$	Subscript for Gas Generator (GG)
$._{H}$	Subscript for ${LH}_{2}$
$._{i}$	Denotes the i-th rocket engine
$._{O}$	Subscript for LOX
**Variables**	
$\omega_{H}$	LH_2_ pump speed [${\mathrm{rad}\;s}^{-1}$]
$\omega_{O}$	LOX pump speed [${\mathrm{rad}\;s}^{-1}$]
$P_{C}$	CC Press. [Pa]
$P_{G}$	GG Press. [Pa]
$P_{H2}$	Sec. line ${LH}_{2}$ Press. [Pa]
$P_{sT}$	Lox tank Press. [Pa]
$P_{TH}$	${LH}_{2}$ Turbi. Press. [Pa]
$P_{TO}$	LOX Turbi. Press. [Pa]
$P_{ull}$	LOX tank ullage Press. [Pa]
$q_{m}$	Main line LOX mass flow [$kg\;s^{-1}$]
$q_{s}$	Sec. line LOX mass flow [$kg\;s^{-1}$]
$q_{CH}$	CC LH_2_ mass flow [$kg\;s^{-1}$]
$q_{CO}$	CC LOX mass flow [$kg\;s^{-1}$]
$q_{GH}$	GG LH_2_ mass flow [$kg\;s^{-1}$]
$q_{GO}$	GG LOX mass flow [$kg\;s^{-1}$]
$q_{so}$	Sec. line outlet LOX mass flow [$kg\;s^{-1}$]
$q_{TH}$	LH_2_ Turbi. mass flow [$kg\;s^{-1}$]
$q_{TO}$	LOX Turbi. mass flow [$kg\;s^{-1}$]
$R_{en}$	Engine thrust reference [N]
$P_{m}$	Main line LOX Press. [Pa]
$P_{s}$	Sec. line LOX Press. [Pa]
RMC	Mixture ratio CC
RMG	Mixture ratio GG
